# Transposable Elements in the Genome of the Lichen-Forming Fungus *Umbilicaria pustulata* and Their Distribution in Different Climate Zones along Elevation

**DOI:** 10.3390/biology11010024

**Published:** 2021-12-24

**Authors:** Francesco Dal Grande, Véronique Jamilloux, Nathalie Choisne, Anjuli Calchera, Gregor Rolshausen, Malte Petersen, Meike Schulz, Maria A. Nilsson, Imke Schmitt

**Affiliations:** 1 Senckenberg Biodiversity and Climate Research Centre (SBiK-F), Senckenberganlage 25, 60325 Frankfurt am Main, Germany; anjuli.calchera@senckenberg.de (A.C.); mcs1989@gmx.de (M.S.); maria.nilsson-janke@senckenberg.de (M.A.N.); imke.schmitt@senckenberg.de (I.S.); 2 LOEWE Centre for Translational Biodiversity Genomics (TBG), Senckenberganlage 25, 60325 Frankfurt am Main, Germany; 3 INRAE URGI, Centre de Versailles, Bâtiment 18, Route de Saint Cyr, 78026 Versailles, France; veronique.jamilloux@inrae.fr (V.J.); nathalie.choisne@inrae.fr (N.C.); 4 Senckenberg Center for Wildlife Genetics, Clamecystrasse 12, 63571 Gelnhausen, Germany; grolshausen@senckenberg.de; 5 Max Planck Institute of Immunobiology and Epigenetics, Stübeweg 51, 79108 Freiburg, Germany; petersen@ie-freiburg.mpg.de; 6 Institut für Ökologie, Evolution und Diversität, Goethe-Universität Frankfurt, Max-von-Laue-Strasse. 9, 60438 Frankfurt am Main, Germany

**Keywords:** TEs, lichens, terrestrial symbiosis, population genomics, environmental gradient

## Abstract

**Simple Summary:**

Accumulating evidence suggests that transposable elements—DNA sequences that can ‘jump’ from one location to another in the genome—may not be randomly distributed in the genome. They might in fact be an important source of adaptive evolution through genome diversification. In this study we present the first in-depth investigation of transposable element content in a lichen-forming fungus. The species we chose is *Umbilicaria pustulata—*an ascomycete that forms symbiotic associations with green algae of the genus *Trebouxia* and is able to inhabit a broad latitudinal and elevational range throughout the European continent. Additionally, we studied the distribution of transposable elements in several populations of the fungus across three mountains in the Mediterranean region. We found several transposable element insertions that display a climate-specific distribution along the elevational gradients. Our study contributes to expanding our understanding of transposable element content and evolution in fungal obligate biotrophs. Particularly, it may serve as a foundation for assessing the impact of transposon dynamics on fungal adaptation to the abiotic environment and the impact of transposon activity on the evolution and maintenance of a symbiotic lifestyle.

**Abstract:**

Transposable elements (TEs) are an important source of genome plasticity across the tree of life. Drift and natural selection are important forces shaping TE distribution and accumulation. Fungi, with their multifaceted phenotypic diversity and relatively small genome size, are ideal models to study the role of TEs in genome evolution and their impact on the host’s ecological and life history traits. Here we present an account of all TEs found in a high-quality reference genome of the lichen-forming fungus *Umbilicaria pustulata*, a macrolichen species comprising two climatic ecotypes: Mediterranean and cold temperate. We trace the occurrence of the newly identified TEs in populations along three elevation gradients using a Pool-Seq approach to identify TE insertions of potential adaptive significance. We found that TEs cover 21.26% of the 32.9 Mbp genome, with LTR Gypsy and Copia clades being the most common TEs. We identified 28 insertions displaying consistent insertion frequency differences between the two host ecotypes across the elevation gradients. Most of the highly differentiated insertions were located near genes, indicating a putative function. This pioneering study of the content and climate niche-specific distribution of TEs in a lichen-forming fungus contributes to understanding the roles of TEs in fungal evolution.

## 1. Introduction

Transposable elements (TEs) are DNA sequences that self-propagate across genomes and are ubiquitous components of almost all prokaryotic [1] and eukaryotic genomes such as plants (e.g., [2,3], fungi [4] and animals [5,6]). Eukaryotic TEs fall into two broad classes: DNA transposons that use a cut-and-paste mechanism for their transposition and retrotransposons that move via a reverse-transcribed RNA intermediate via a copy-and-paste mechanism. TEs can be further classified into superfamilies and families based on specific sequence features [7,8,9]. Most TEs present in eukaryotic genomes are genomic fossils, i.e., inactive remnants of once-active copies [10,11]. Their variation in copy number and size is responsible for much of the large differences in genome size observed even among closely related species [12,13,14]. On the other hand, the most recent, likely active, transposable fraction of the mobilome—all repeated sequences except microsatellites—remains silenced under normal conditions. TEs are activated by ontogenetic factors and/or environmental cues [15,16]. By their repetitive nature, TEs provide hotspots for ectopic (non-homologous) recombination and induce chromosomal rearrangements as well as gene shuffling, leading to loss of genomic portions or expansion of gene copy numbers. Being mobile, TEs can further be located in coding or regulatory regions, thus strongly affecting gene expression and gene structure and/or function. TEs can thus passively and actively impact genome plasticity and extensively shape eukaryotic genome evolution [17,18].

TEs generate evolutionary novelty and respond to environmental change, indicating that they are likely to play a relevant role in adaptation [19,20,21,22,23,24,25]. The relationship between TEs and environmental adaptation is complex, as both activation and repression of transposition in response to environmental changes have been reported [26,27,28]. Most TEs remain silent and evolve in a neutral fashion, while only a minor fraction have adaptive roles (e.g., [29]). Several studies have suggested that the presence of a certain number of potentially active TEs may increase the genome’s ability to cope with environmental stress in a variety of ways, e.g., via major genomic rearrangements [30], TE-driven creation of new regulatory networks involving genes in the TEs’ proximity [31,32,33,34] and/or genome alteration via newly generated TE copies [35]. As such, TEs can be a major source of intra-population genetic variation in response to environmental pressures (e.g., [36,37]). For instance, TE composition and/or copy number variation in response to micro-climatic conditions was reported for natural populations of wild barley, *Arabidopsis thaliana* [9,38], *A. arenosa* [39] and several Brassicaceae species [40]. However, there is a general lack of understanding on how environment influences TE abundance and the activity of most TEs in most non-model species. The range and phenotypic consequences of the heritable mutations produced through TE mobilization remain largely unknown.

Fungi are a diverse group of organisms colonizing all habitats on Earth [41,42]. Their remarkable diversity in terms of morphologies, lifestyles, genome sizes, reproductive modes and ecological niches makes them an ideal group for comparative genomics. Due to their relatively small genome size compared to plants and animals (e.g., 37 Mbp on average in Ascomycota and 46 Mbp in Basidiomycota; [43]), fungal genomes are easier to assemble and annotate. The past decade has seen an extraordinary increase in fungal genomic research and also in the area of TE research. The increased availability of high-quality assemblies for a large number of fungi has enabled kingdom-wide comparative studies [4,44]. The TE content of fungal genomes is variable, typically ranging from 0 to 30%, with up to 90% in the plant–pathogen *Blumeria graminis* [45,46]. Retrotransposons with long terminal repeats (LTR) are the most abundant TEs in fungal genomes [47]. Several studies have shown that TEs are a major driving force for adaptive genome evolution in fungi [48], especially in fungal plant–pathogens [44,49]. In fact, animal-related and pathogenic fungi tend to have more TEs inserted into genes than fungi with other lifestyles and may play an important role in effector gene diversification [50,51]. Surprisingly, lichen-forming fungi, a group of highly diverse, ecologically obligate biotrophs, have been completely neglected in TE research. Lichens are textbook examples of ecologically successful symbioses, being the result of a tightly integrated relationship between a fungus, typically an ascomycete, and green algae and/or cyanobacteria [52]. Lichens, due to their ability to tolerate environmental extremes, their specialized nutritional mode involving more or less strictly selected photosynthetic symbionts and their varied morphologies and modes of reproduction represent an important missing piece of the puzzle in our attempt to understand the impact of TE activity on the evolutionary trajectory and architecture of fungal genomes.

Here we provide the first in-depth report on the abundance and distribution of TEs in the genome of a lichen-forming fungus, the ascomycete *Umbilicaria pustulata* (L.) Hoffm. [53]. *U. pustulata* is a widespread macrolichen that grows attached to rocks from southern Europe to northern Scandinavia. Population genomics analyses revealed the presence of otherwise morphologically indistinguishable ecotypes in *U. pustulata*, i.e., intra-specific lineages, differentially adapted to the Mediterranean and cold temperate climate zone, and interacting with different algal symbiont communities [54,55]. The availability of a high-quality, PacBio-based reference assembly [56], together with marked genome-wide climatic niche differentiation data [54] and the possibility to sample this widespread and abundant species along replicated elevation gradients, make *U. pustulata* an ideal model to study the TE content of a lichen-forming fungal genome and its potential link to intra-specific adaptive variation. Specifically, we asked the questions: (i) How diverse is the mobilome in *U. pustulata*?; (ii) To what extent does TE abundance vary between populations and across gradients?; (iii) Are there ecotype-specific TE insertions, and if so, where are they located in the genome? To address these questions, we tracked the insertion frequencies of the newly annotated TEs in populations representing the Mediterranean and the cold temperate ecotypes of the species. To disentangle general trends from local differentiation, we sampled populations across three elevational gradients each encompassing the Mediterranean and the cold temperate climate zone.

## 2. Materials and Methods

### 2.1. The Genome of U. pustulata

We used the genome assembly by Greshake Tsovaras et al. [56] as reference for TE prediction and annotation (accession GCA_008636195, BioProject: PRJNA464168). The haploid genome of *U. pustulata* is 32.9 Mbp long, with 43 scaffolds and an N50 length of >1.8 Mbp.

### 2.2. Pool-Seq Sequencing of 15 U. pustulata Populations

To predict the copy insertion frequencies at TE loci across three elevational gradients, we used whole-genome sequencing data from pools of individuals from 15 natural lichen populations (100 lichen thalli per population). The 15 pools were collected along three elevational gradients in Southern Europe, i.e., Mount Limbara (Sardinia, Italy; 6 populations, IT), Sierra de Gredos (Sistema Central, Spain; 6 populations, ESii) and Talavera-Puerto de Pico (Sistema Central, Spain; 3 populations, ESi) (Table 1), as described in [54]. Individuals were pooled in equimolar concentrations and each pool was sequenced on an Illumina HiSeq platform (2 × 100 bp for IT and ESi; 2 × 150 bp for ESii). The Pool-seq data was quality-filtered using Trimmomatic v0.39 [57] with a length cutoff of 80 bp and a quality cutoff of 26 in a window of 5 bp. Reads with Ns were removed and an additional quality trimming using a modified Mott algorithm was performed using the script trim-fastq.pl from the PoPoolation v1.2.2 pipeline [58]. After trimming, the sequencing depth varied between 24.3 and 37.3 million paired-end reads (Table 1).

### 2.3. De Novo TE Prediction: Building a U. pustulata TE Consensus Library

We used the TEdenovo pipeline from the REPET package v2.5 [59,60] to generate a TE consensus library in *U. pustulata*. Briefly, the pipeline was used to perform a self-alignment of the reference genome to detect repeats, to cluster the repetitions and to perform multiple alignments from the clustered repetitions to create consensus TE sequences. Consensus TEs were subsequently classified using the PASTEClassifier pipeline v2.0 [61], which follows Wicker’s classification [7] using structural and homology-based information (i.e., terminal repeats, poly(A) tails, ORFs, tandem repeats, etc.) and the following databases: ‘repbase20.05_ntSeq_cleaned_TE.fa’, ‘repbase20.05_aaSeq_cleaned_TE.fa’ and ‘ProfilesBankForREPET_Pfam27.0_GypsyDB.hmm’ (https://urgi.versailles.inra.fr/download/repet (accessed on 9 January 2018)). We set the minNbSeqPerGroup parameter to 3 (i.e., 2n + 1) because *U. pustulata* is haploid. All remaining parameters used for these analyses can be found in the TEdenovo and TEannot configuration files (Additional Files 1, 2).

We then performed extensive automated as well as manual curation of the TE consensus library to minimize redundancy as well as false positives. For this purpose, we first performed a two-step annotation [62] on contigs longer than 5 Kbp, i.e., 1st round: steps 1 —taking all matches found by BLASTER, RepeatMasker and CENSOR, 2—normal and random, 3—using Grouper, Recon and Piler as clustering methods and 7—removing duplicated/spurious fragments and applying the long join procedure for nested copies of TEs identified by the TEannot pipeline part. We only retained TE consensus sequences having at least one full-length copy (FLC, i.e., length of fragments between 95% and 105% of consensus length) to build the final TE library. This was followed by a 2nd round consisting of TEannot steps 1, 2, 3, 4, 5, 7 and 8, using the final TE library to annotate the genome.

Finally, we performed a copy divergence analysis of TE classes based on Kimura distances by calculating Kimura 2-parameter divergence [63] between each TE copy and its consensus sequence using the utility scripts provided in the RepeatMasker package. These were also used to construct a TE landscape divergence plot by grouping copies within TE superfamilies and calculating the percentage of the genome occupied by each TE superfamily.

### 2.4. Evaluation of TE Copy Insertion Frequencies across the Different U. pustulata Populations

We used the PoPoolationTE2 v1.10.04 pipeline [64] to compute population-wide TE copy insertion frequencies of the curated TE library across the 15 populations described above. For this, we performed a ‘joint’ analysis using both quantitative and qualitative information extracted from paired-end reads mapping on the TE-annotated reference genome and a set of reference TEs to detect TE copy insertion frequencies in populations. Frequency values in this case correspond to the proportion of individuals in a population for which a TE copy is present at a given locus.

We used the curated *U. pustulata* TE library and the *U. pustulata* reference genome described above to produce the ‘TE-merged’ reference file (available at: 10.6084/m9.figshare.14784579 (accessed on 8 November 2021)) and the ‘TE-hierarchy’ file (Additional File 3) as follows. Sequences corresponding to the TE annotations were extracted to GFF3 format using the command ‘gff3_compulsory_match_part: yes’ in TEannot and masked in the reference genome using the tools getfasta and maskfasta from the BEDTools suite [65], respectively. The resulting TE sequences were concatenated with the masked genome to form the ‘TE-merged’ reference. For every TE copy we also retrieved TE sequence name, family, and order to build the required ‘TE-hierarchy’ file. For each *U. pustulata* pool, we mapped forward and reverse reads separately against the ‘TE-merged’ reference using the local alignment algorithm BWA-SW v0.7 [66] with default parameters. The obtained SAM alignment files were then converted to BAM files using samtools view v1.9 [67]. Paired-end information was restored from the previous alignments using the se2pe (--sort) tool from PoPoolationTE2 v1.10.04. Using the ppileup tool from PoPoolationTE2 we then created a ppileup file (--map-qual 15) that summarizes, for every base of the genome, the number of PE reads spanning the site—i.e., physical coverage—as well as the structural status inferred from the paired-end reads covering the site (i.e., indicating whether one or both boundaries of a TE insertion are supported by significant physical coverage).

Heterogeneity in physical coverage among populations may lead to discrepancies in TE frequency estimation and in a substantial fraction of sample-specific insertion false positives [64]. Hence, to reduce the number of false positives, we normalized the physical coverage across the *U. pustulata* populations via a subsampling and a rescaling approach: In order to balance the loss of information with the homogeneity of the TE frequency, we used the stat-coverage tool from PoPoolationTE2 to obtain information on the physical coverage in our dataset. We then used the subsamplePpileup tool (--target-coverage 16) to discard positions with a physical coverage below 16x and rescale the coverage of the remaining sites to that value.

We identified signatures of TE polymorphisms from the previously subsampled file using the identifySignature tool following the joint algorithm (--mode joint; --min-count 3; --signature-window minimumSampleMedian; --min-valley minimumSampleMedian). Then, for each identified site, we estimated TE frequencies in each pool using the frequency tool. Eventually, we paired up the signatures of TE polymorphisms using pairupSignatures tool (--min-distance 100; --max-distance 500), yielding a final list of TE loci in the reference genome with their frequencies for each pool. Each TE insertion was manually checked using IGV v2.5 [68]. TE loci predictions with unusually high read coverage, i.e., resulting from spurious alignments to unmasked repeats, were discarded from further analysis. The stringent filters applied here, together with the inability of PoPoolationTE2 to detect nested TEs [64], may lead to an underestimation of TE activity across *U. pustulata* populations. On the other hand, such a conservative approach almost certainly eliminates false insertions.

TE loci supported by significant physical coverage were considered polymorphic if they had a frequency difference of at least 0.05% among populations. TE loci with frequencies ≥0.95% were considered fixed in the populations. The similarity of populations based on their TE composition was investigated using nonmetric multidimensional scaling (NMDS) on all detected TE insertion frequencies using the function metaMDS from the vegan package [69] for R [70].

### 2.5. Identification of TE Loci Significantly Differentiated between U. pustulata Ecotypes

To identify highly differentiated TE loci (hdTEs) between *U. pustulata* ecotypes we performed a differential abundance analysis using the microbiomeSeq [71] and DeSeq2 [72] R packages. For this purpose, we contrasted the normalized relative abundances of all TE copy insertions in DeSeq2 to detect differentially abundant TE copy insertions (at α = 0.01) between populations representing the Mediterranean (populations IT1-4, ESii1 and ESi1) and the cold temperate (IT6, ESii3-6 and ESi2-3) ecotypes. From the analysis we excluded populations IT5 and ESii2, because they represent admixed populations of both ecotypes [54].

### 2.6. Functional Characterization

To identify genes potentially impacted by TE insertions, i.e., genes overlapping with TEs or in the proximity of TEs (1 kbp up or downstream from each TE insertion), we cross-referenced the TE annotation file with the gene annotation file [56] using the *intersect* tool of the BEDTools suite [65].

### 2.7. Population Structure Based on Genome-Wide SNPs

Population structure based on genome-wide single-nucleotide polymorphisms (SNPs), i.e., the positional relations among populations based on their genetic distances, was detected by analyzing pairwise quantile distance matrices (0.975, 0.75, 0.5, 0.25 and 0.025) based on the pairwise fixation index (F_ST_) among all populations using a three-way generalization of classical multidimensional scaling (DISTATIS; [73]). Briefly, we used the sorted, duplicate-removed BAM files of reads mapped to the *U. pustulata* reference genome. High-quality (i.e., after removing duplicated reads and genomic indels) SNPs were called using SAMtools mpileup and normalized to a uniform coverage of 30 across all populations with PoPoolation2 [74]. For this we used the synchronized mpileup file (i.e., ‘sync’ file containing the allele frequencies for every population at every base in the reference genome) and the script subsample-synchronized.pl (--without-replacement), excluding positions with a coverage exceeding the 2% of the empirical coverage distribution of each pool. Genetic differentiation (F_ST_) was calculated with fst-sliding.pl in PoPoolation2 on the subsampled sync file (F_ST_ dataset available at: 10.6084/m9.figshare.14784579 (accessed on 8 November 2021)). We only considered SNPs with a minimum read count of 4 and a minimum mapping quality of 20. A more detailed description of the methods can be found in [54].

## 3. Results

### 3.1. TE Landscape in U. pustulata

The mobilome spans 21.26% of the *U. pustulata* genome length (Table S1). We annotated 119 TE consensus sequences (available via the INRAE data repository: https://doi.org/10.15454/KXPSUY (accessed on 13 November 2021) and at: 10.6084/m9.figshare.14784579 (accessed on 8 November 2021)) for a total of 5956 TE copies (704 of which are full-length) and 6758 TE fragments, for a cumulative coverage of 6,996,427 bp (Table 2, Tables S1 and S2). Retrotransposons (Class I) cover 15.6% of the genome of *U. pustulata*, while DNA transposons (Class II) cover 3.5%. Among the Class I elements, Gypsy are the most represented (8.8% of the genome), followed by Copia elements (4.1%). Helitron are the most abundant elements within the Class II elements (1.7%), followed by terminal inverted repeats (TIR; 1.2%).

**Figure 1:**
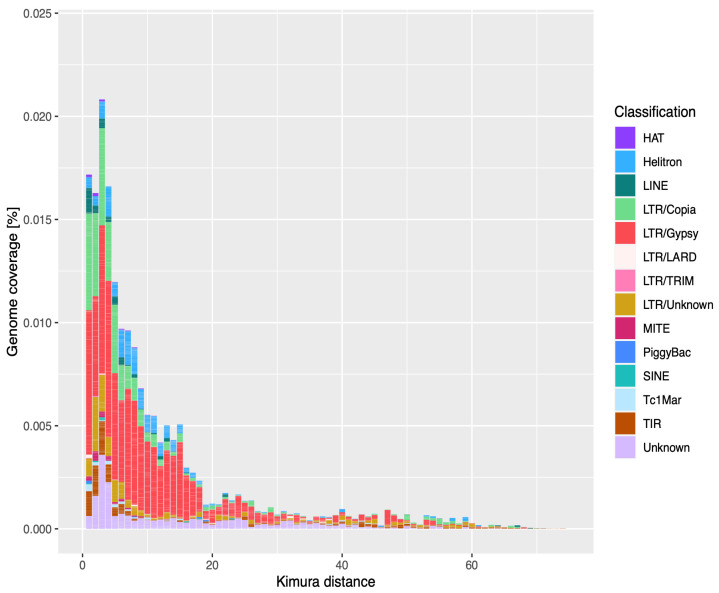
Repeat landscape plot in *U. pustulata*. Sequence divergence of each TE copy from the corresponding consensus sequence was measured by Kimura (K2P) distance. The further to the left a peak in the distribution, the younger the corresponding TE fraction generally is.

TE copies have a median nucleotide identity of ~90% with their respective TE family consensus sequence, ranging from 88.7% for Helitron (Class II) and 86.2% for LTR elements (Class I) to 95.3% for PiggyBac (Class II) and 94% for LINE elements (Class I). The distribution of TE copies’ identities to their family consensus sequences suggests recent activity (Figure 1, Table S3).

### 3.2. TE Variation across U. pustulata Populations

We used the PoPoolationTE2 pipeline [64] on the *U. pustulata* reference genome [56] to detect variations in TE frequencies in 15 natural populations across three replicated elevational gradients.

After manual curation, we retained 182 TE loci belonging to 12 superfamilies with a minimum physical coverage of 16 (Table 3 and Table S4). Of these, 68 insertions were fixed across populations, i.e., they had a minimum frequency of 0.95 within each population. Copia elements were the most frequently detected loci, representing 43% (49 loci) of all polymorphic insertions, followed by TIR elements (19.3%, 22 loci) (Table 3B). 

**Figure 2:**
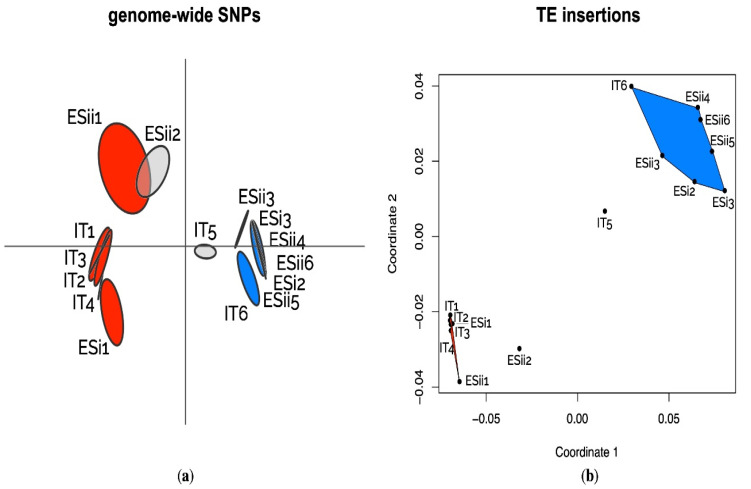
(**a**) Pattern of genetic structure among populations based on pairwise F_ST_ genetic distances calculated on 447.470 polymorphic SNPs. (**b**) Nonmetric multidimensional scaling (NMDS) ordination plot illustrating population structure based on TE copy insertion frequencies in 15 populations of *U. pustulata*. IT: Italian gradient; ES: Spanish gradients (i, ii). The populations from Mediterranean climate (red) and cold temperate climate (blue) form clusters (with the exception of IT5 and ESii2, which have intermediate positions).

We further compared population structure based on 447,470 genome-wide SNPs (dataset available at: 10.6084/m9.figshare.14784579 (accessed on 8 November 2021)), with the population divergence based on the variations of TE frequencies across populations. Both SNP-based and TE frequency-based ordinations show that populations can be grouped into two clearly distinct clusters corresponding to the Mediterranean and cold temperate ecotypes of the lichen-forming fungus *sensu* [54] (Figure 2).

### 3.3. Variations of TE Frequencies between Ecotypes

We identified TE loci that were highly differentiated (hdTEs) between the two ecotypes because these loci might represent differential fixation/loss between ecotypes and have particular functional relevance. We identified 28 hdTEs (Table 3C). Of these, seven were exclusively found in the cold temperate populations, 19 showed significantly higher frequency in the cold temperate populations and one was more abundant in the Mediterranean populations (a short Copia11 fragment in scaffold9_123163). One Copia element was almost exclusively found in the two Spanish gradients (an almost full-length Copia11 copy in scaffold9_1443709). This insertion was absent in the Mediterranean climatic zone and linearly increased in abundance with elevation (Figure S1, Table S4).

The analysis of hdTEs between ecotypes showed an overrepresentation of Copia elements (16 loci, 57.1%). Among hdTEs we also found four TIR, three Helitron, three unknown, one MITE and one PiggyBac element. Compared to all other TE insertions detected across populations, hdTEs were significantly more similar to their consensus sequence (Wilcoxon signed rank sum test with *p* < 0.0001 both in terms of sequence identity and length coverage). Eighteen hdTEs displayed sequence identity and length coverage towards their respective consensus sequence greater than 95%.

### 3.4. Potential Functional Impact of TE Insertions

One hundred and two out of 114 polymorphic TE loci were inserted either inside a gene (27 TE loci, 25 in coding positions) or in a possible regulatory region (in the 1-kb region surrounding a gene). These include all except two hdTEs (Tables S3 and S4).

## 4. Discussion

### 4.1. The U. pustulata Mobilome

In this work we studied the content of transposable elements in the genome of the lichen-forming fungus *U. pustulata*. Furthermore, we analyzed the variation in TE insertion frequency in populations representing two ecotypes distributed along three gradients spanning the elevational range of the species, i.e., from the Mediterranean to cold temperate climate zones.

The repeat content in *U. pustulata* of 21% is rather high compared to the repetitive content in other fungal genomes, which typically ranges from 0 to 30% [47,75]. It is also higher than the predicted 15% TE content in another lichen-forming fungus, the Eurotiomycete *Endocarpon pusillum* [76]. The *U. pustulata* TE landscape is particularly rich in retrotransposons (class I), especially the LTR retrotransposons Gypsy and Copia. This is a general feature in fungi [47]. The Class I/Class II genomic coverage ratio of 1.56 is in line with what has been reported for Ascomycetes (0.78–4.23; [47]).

A substantial portion of the annotated TE copies are highly similar to their consensus, which is often interpreted as a signature of rapid and recent bursts of TE activity in the genome (e.g., [77]). Some TE families, such as Gypsy, on the other hand, displayed a broader range of identity rate with their consensus, suggesting slower colonization of the *U. pustulata* genome with these elements. In the absence of a molecular clock for *U. pustulata*, it is, however, difficult to precisely evaluate the time when the TE bursts possibly occurred and how much time it took for the TEs to spread in the genome.

Population-level analyses of TE insertion frequencies in 15 populations of *U. pustulata* along three elevational gradients showed that a substantial part of the TEs can be considered stable and fixed among populations. The clustering of populations based on the detected TE loci between ecotypes recapitulated almost exactly the population divergence based on genome-wide SNPs. This suggests that TE variation is mainly a result of drift between populations. The predominant evolutionary neutrality of TE variation has already been reported for other groups of organisms, such as nematodes [78], and other fungi [79].

### 4.2. Ecotypic Differentiation Patterns of TE Insertions and Their Potential Functional Impact

Although adaptive TE insertions may be marginal compared to the overall mobilome dynamics [79], it is broadly recognized that TEs can play important regulatory roles and may contribute substantially to adaptive evolution in a variety of organisms [24,26,80,81]. To identify TE insertions likely linked to climatic niche, we studied loci where the TE frequencies were significantly differentiated by fungal ecotype, recurrently across the gradients (hdTEs). Overall, the high similarity of hdTEs to their consensuses and the high variability in insertion frequency among populations—some of which linearly correlated with elevation (Copia11 in scaffold9_1443709; Copia8 in scaffold3_1523900) or absent from some of the gradients (Copia05 in scaffold24_266994)—suggest that most of the hdTEs have recently been active in *U. pustulata* and are possibly still active, in particular in populations located in the cold temperate climate zone.

Copia retrotransposons are the younger, most active elements of the *U. pustulata* mobilome. When Copia elements are in proximity of a gene, their regulatory role is typically exerted via regulation of gene expression by small RNAs, whereas when inserted within genes they can give rise to alternative splice variants [38,82]. Genome expansion related to retrotransposon amplification has been shown to occur in plants as a result of environmental adaptation (e.g., [83,84]). Global transcriptomic responses of Copia elements have been linked to heat stress in *Arabidopsis* spp. [40] and to various environmental stresses in *Eucalyptus* [85].

The identified hdTEs are prime candidates for future functional validation, e.g., via targeted transcriptomic and proteomic analyses, to test whether and how they influence adaptation of the lichen ecotype to different climatic niches. Particularly interesting in this regard could be the effects of TEs inserted near (i) genes involved in cell wall biosynthesis: a Copia element near a putative GPI ethanolamine phosphate gene, controlling membrane-to-cell wall transfer of fungal adhesins by membrane-anchored transglycosidases [86]; a TIR element near *Sac7*, a known activator of the small GTPase RHO1, which plays an essential role in the control of cell wall synthesis and organization of the actin cytoskeleton [87]; (ii) genes involved in nutrient assimilation: a Copia element near a NADP-specific glutamate dehydrogenase, a key enzyme in the assimilation of alternative nitrogen sources through ammonium [88]; an Helitron element near an acid protease, whose secretion grants access to the carbon and mineral nutrients within proteins in the cells of the plant host in fungal endophytes [89]; an unknown TE element inserted near inositol-pentakisphosphate 2-kinase, an enzyme involved in the decomposition of organic phosphates whose activity is modulated by environmental pH [90]; (iii) genes involved in DNA repair mechanisms: a Copia element near a putative DNA glycosylase, a gene involved in single-base excision repair mechanisms [91]; (iv) genes involved in reproduction and environmental sensing: an unknown TE element located near a conidiation-specific gene, which plays a role in balancing asexual and sexual development, a process regulated by several factors including light, temperature, humidity and nutrient availability [92,93]; (v) genes involved in secondary metabolism: a PiggyBac element within a type-I polyketide gene cluster containing fixed nonsense mutations in its core biosynthetic gene only at high elevations (i.e., in the cold temperate climate zone) [94]. TEs have been previously identified as regulators of biosynthetic gene clusters in ascomycetes: the lower expression of the penicillin cluster in *Aspergillus nidulans* in the absence of *Pbla* element is a typical example [95].

### 4.3. Outlook and Future Perspectives

To our knowledge, this is the first in-depth report on a lichen mobilome, based on a highly contiguous and complete PacBio-based reference assembly. As more consensus TE libraries become available in the future as a result of improved sequencing and assembling technologies, the study of the mobilome of lichen-forming fungi will contribute key insights to the understanding of TE evolution, in particular in the following research areas:

(1) Role of reproductive mode on TE abundance and composition: the dynamics in TE load according to the reproductive modes are still a matter of debate. Theoretically, sexual reproduction may either facilitate TE accumulation by providing a means of spreading to all individuals in a population or restrain TE accumulation via purifying selection [96]. On the other hand, TE movements may constitute an important source of genome plasticity compatible with adaptive evolution in predominantly asexual species [78]. Broad-scale comparative analyses of different sexual and asexual lineages in both nematodes and arthropods revealed no evidence for differences in TE load according to the reproductive modes [97,98]. Lichens are ideal study systems to address this question, as congeneric, closely related species often differ strikingly in their modes of reproduction [99,100]. In our case, the sister species of the predominantly asexual *U. pustulata*, *U. hispanica*, reproduces mainly via sexual ascospores [101,102].

(2) Link between TE content and fungal life strategies: TE count tends to be elevated in fungal plant symbionts [103]. This is because recurrent adaptation to symbiosis seems to involve relaxed genome control against duplications, TE proliferation and overall growth in genome size [81]. About half of the currently described ascomycete species are involved in a lichen symbiotic association. This symbiotic lifestyle is believed to have arisen independently on several occasions in the evolution of Ascomycota [52]. Comparing the mobilome of several unrelated lichen-forming fungi across the Fungi will provide important basal information to understand the evolutionary consequences of the symbiotic lifestyle on the fungal mobilome.

(3) Intra-specific variation and role of TEs in adaptive evolution: several studies have shown that TE insertion patterns may differ between closely related fungal species occupying different niches (e.g., *Ustilago maydis* and *Sporisorium scitamineum* [104]), or even between strains within the same species (*Magnaporthe grisea* [105]). Many lichen species are characterized by wide ecological amplitudes, with distributional ranges spanning multiple climate zones. Furthermore, long-lived sessile organisms such as lichens are more likely to experience strong selective pressures resulting in particularly abrupt genetic breaks between differentially selected populations over short distances [54,106]. Lichens are therefore ideal systems to test the intra-specific differentiation in TE content and its potential role in affecting host fitness in different environments.

(4) TE content in lichen-associated photobionts: Nearly 40 genera of green algae (~100 species) have been reported from lichen symbioses. Studies on the TE content of green algae are scarce. While the TE abundance seems to be low in the green algal lineage [107,108], TEs may have important functional roles. For instance, TEs may have considerably contributed to gene regulatory sequence evolution in the green algal model species *Chlamydomonas reinhardtii* [108]. TEs were reported as the major driver of chromosome specialization in two out of the twenty chromosomes in the marine algal *Ostreococcus tauri*, the smallest free-living eukaryote, possibly contributing to environmental niche adaptation and modulation of reproduction [109]. Lichen photobionts are an interesting and highly diverse group of unicellular eukaryotes to study in relation to TE diversity and evolution, especially considering the high symbiotic specificity, the high intra-specific diversity and strong environmental structuring found in many taxa [110,111,112,113,114].

In summary, our pioneering study of TE content and variation of a lichen-forming fungus provides valuable baseline data for future investigations. It opens up new perspectives for targeted analyses of the potential effect of TE dynamics on the evolution, fitness and adaptability of *U. pustulata* and more generally lichen-forming fungi and of other symbiotic systems.

## Figures and Tables

**Table 1:** Population IDs, coordinates, elevations and Pool-seq read numbers for 15 *U. pustulata* populations along three elevational gradients.

Country	Population ID	Lat	Long	Elevation m a.s.l.	No. Paired-End Read #	Mean Read Length
Italy	IT1	40.7577	9.0794	176	29,162,770	99.3
	IT2	40.7778	9.0546	297	28,279,628	99.3
	IT3	40.8503	9.1119	588	26,570,943	99.4
	IT4	40.8568	9.1340	842	31,720,828	99.4
	IT5	40.8573	9.1642	1125	31,755,901	99.4
	IT6	40.8524	9.1732	1303	32,064,853	99.4
Spain 1	ESii1	40.2028	−5.2334	706	26,758,269	141.8
	ESii2	40.2069	−5.2327	887	24,295,101	141.7
	ESii3	40.2116	−5.2337	1082	29,236,274	141.9
	ESii4	40.2183	−5.2335	1258	33,333,561	141.6
	ESii5	40.2253	−5.2375	1480	24,672,545	141.7
	ESii6	40.2322	−5.2389	1699	26,690,508	141.5
Spain 2	ESi1	39.9946	−4.8679	477	28,862,057	99.5
	ESi2	40.2899	−4.9927	859	37,303,042	99.5
	ESi3	40.3230	−5.0173	1417	35,351,050	99.5

**Table 2:** Explanation of A and B.

**A.** Summary of Class I and II TE elements found in the *U. pustulata* genome.											
**Class**		**Total Length**		**No. Copies**		**No. Full Length Copies**		**Median Identity ^1^**		**Median Length**	
Class II		1,146,170		1863		156		91.4		657.9	
Class I		5,118,614		2902		465		90.3		1162.5	
unknown		731,643		1191		83		88.1		323.4	
**B.** Summary of TE elements subdivided into superfamilies for the *U. pustulata* genome.											
**Class**	**Order**	**Superfamily**	**No. Elements**	**Total Length**	**No. Copies**		**No. Full Length Copies**		**Median Identity ^1^**		**Median Length**
Class II	DHX	Helitron_01	7	553,513	680		23		88.7		498.6
	DTA	HAT	1	24,206	80		4		89.98		186.5
	DTB	PiggyBac	1	12,236	10		4		95.3		1481.0
	DTT	Tc1Mar	4	104,574	139		28		89.6		1029.4
	DTX	TIR	18	380,415	824		86		92.0		648.2
	DXX	MITE	4	71,226	130		11		93.0		521.0
Class I	RII + RIX	LINE	5	317,234	155		33		94.0		923.1
	RLC	Copia	25	1,333,809	865		166		92.0		1350.2
	RLG	Gypsy	23	2,904,582	1296		215		89.8		1246.0
	RLX	LTR	15	538,504	550		46		86.2		942.6
	RXX	LARD	1	20,415	25		1		816.6		383.0
	RXX	TRIM	1	4070	11		4		96.8		126.0
	No	Unknown	14	731,643	1191		83		88.1		323.4
total			119	6,996,427	5956		704		147.1		743.0

^1^ Identity = % sequence similarity between TE copy and the respective consensus sequence.

**Table 3:** Explanation of A–C.

**A.** TE copy insertion in 15 populations of *U. pustulata* (min. physical coverage: 16×).		
**TE Family**	**Copy No.**	**%**
Copia	62	34.1
TIR	31	17.0
Unknown	23	12.6
Helitron	22	12.1
Gypsy	16	8.8
LTR	10	5.5
MITE	8	4.4
LARD	5	2.7
TC1Mar	2	1.1
HAT	1	0.5
LINE	1	0.5
Piggybac	1	0.5
**B.** Polymorphic TE copy insertion in populations.		
**TE Family**	**Copy No.**	**%**
Copia	49	43.0
TIR	22	19.3
Unknown	13	11.4
Helitron	10	8.8
Gypsy	5	4.4
LTR	5	4.4
MITE	5	4.4
LARD	1	0.9
TC1Mar	2	1.8
HAT	1	0.9
Piggybac	1	0.9
**C.** hdTEs between *U. pustulata* ecotypes.		
**TE Family**	**Copy No.**	**%**
Copia	16	57.1
TIR	4	14.3
Helitron	3	10.7
Unknown	3	10.7
MITE	1	3.6
PiggyBac	1	3.6

## Data Availability

The datasets supporting the conclusions of this article are available in the Figshare repository, https://doi.org/10.6084/m9.figshare.14784579 (accessed on 8 November 2021).

## Supplementary Materials

The following are available online at https://www.mdpi.com/article/10.3390/biology11010024/s1. Figure S1: Insertional pattern of Copia 11 in scaffold9:1443709 along elevation, Table S1: Description of each TE fragment in the *U. pustulata* genome (‘match_part’ output from TEannot), Table S2: TE fragments bed file, Table S3A: TE loci significantly differentiated between *U. pustulata* ecotypes (hdTEs) and genes in their proximity (1 Kbp up or downstream from TE insertion) and Table S3B: Non-differentiated TE loci between *U. pustulata* ecotypes and genes in their proximity, Table S4: TE insertion frequencies in 15 natural populations across three replicated elevational gradients, Additional File 1: TEdenovo parameters, Additional File 2: TEannot parameters and Additional File 3: TE hierarchy file.
