# Supplementary figures and images for: Transposable Elements in the Genome of the Lichen-Forming Fungus Umbilicaria pustulata and Their Distribution in Different Climate Zones along Elevation
Source: Biology (Basel). 2021 Dec 24;11(1):24. doi: 10.3390/biology11010024 (PMC8773270; doi:10.3390/biology11010024)

# Insertional pattern of Copia 11 in scaffold9:1443709 along elevation

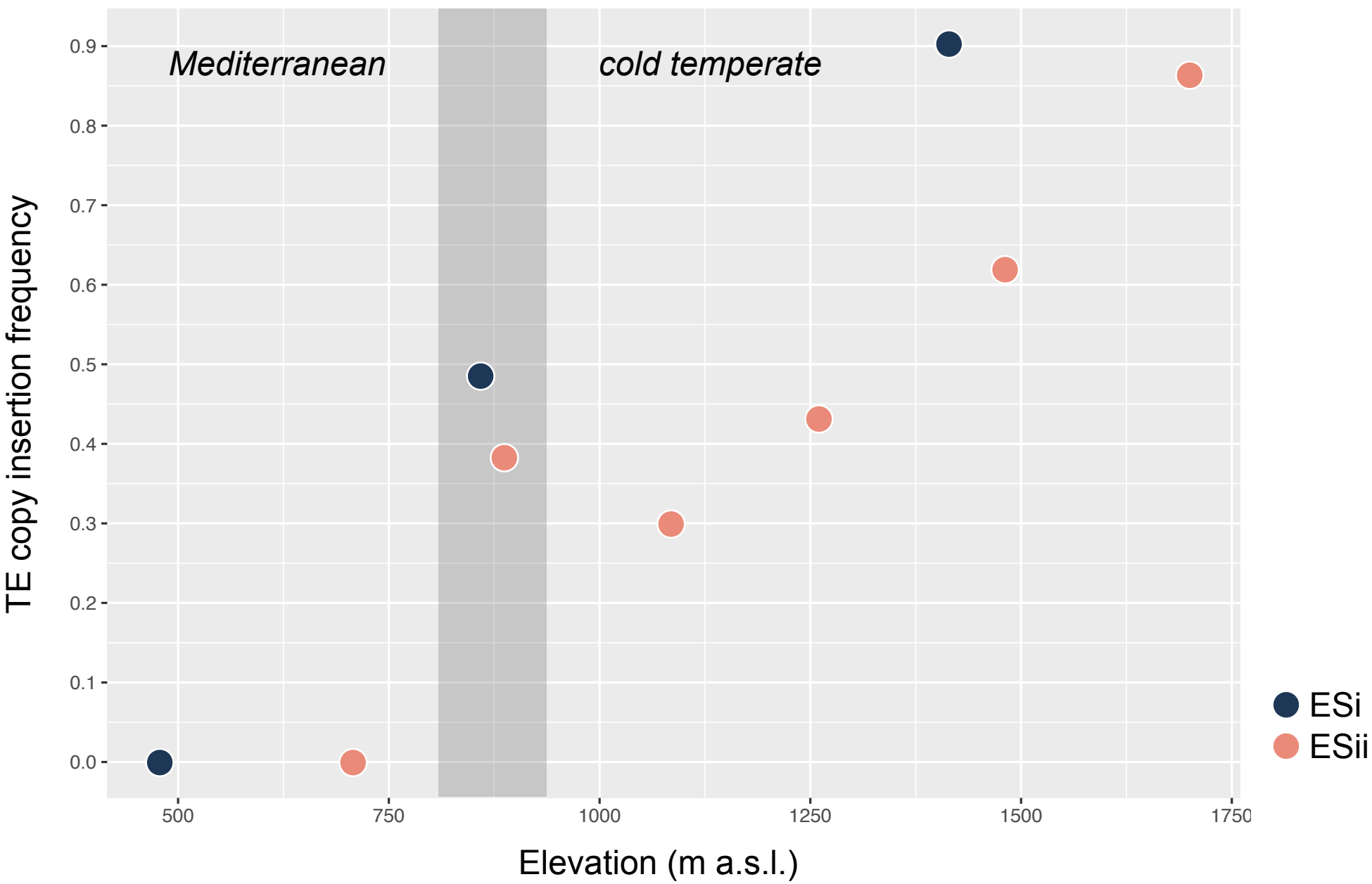

Supplement: Supplementary file 1 [file biology-11-00024-s001.zip › Supplementary_Materials/Figure_S1.pdf]
